# Jackson’s Parrot: Samuel Beckett, Aphasic Speech Automatisms, and Psychosomatic Language

**DOI:** 10.1007/s10912-015-9375-z

**Published:** 2016-02-27

**Authors:** Laura Salisbury, Chris Code

**Affiliations:** Department of English and Film, University of Exeter, Room 221, Queen’s Building, Queen’s Drive, Exeter, EX4 4QH UK; Department of Psychology, University of Exeter, College of Life and Environmental Sciences, Washington Singer Building, Perry Road, Exeter, EX4 4QG UK

**Keywords:** Samuel Beckett, aphasia, John Hughlings Jackson, speech automatisms, language, neuroscience

## Abstract

This article explores the relationship between automatic and involuntary language in the work of Samuel Beckett and late nineteenth-century neurological conceptions of language that emerged from aphasiology. Using the work of John Hughlings Jackson alongside contemporary neuroscientific research, we explore the significance of the lexical and affective symmetries between Beckett’s compulsive and profoundly embodied language and aphasic speech automatisms. The interdisciplinary work in this article explores the paradox of how and why Beckett was able to search out a longed-for language of feeling that might disarticulate the classical bond between the language, intention, rationality and the human, in forms of expression that seem automatic and “readymade”.

In the June of 1845 a parrot, known simply as Poll, was ejected from a funeral. The bird had been taught to swear by the deceased, one Andrew Jackson, who also happened to be the 7th President of the United States. Poll was removed because as the preacher spoke to those gathered in the congregation, the bird began to squawk such unpretty interruptions that the work of the funeral could not proceed. An embarrassing situation, certainly, but there is little sense that the parrot was ultimately deemed to be at fault. Despite the seemingly inevitable suspicion that this uncanny bird may have been cannily up to something, it is likely that those in attendance would have taken a Cartesian view of the situation. In *Discourse on the Method* of 1649, René Descartes influentially determined that it was the capacity for propositional speech that separated the human from the animal or from the mechanical that, in fact, animals simply were. Talking parrots proved no problem for Descartes’s logic, for they were deemed simply to be fleshly machines that uttered in ways that were to be firmly distinguished from the speech proper to humanity. As Descartes puts it:

…we can see that magpies and parrots can utter words as we do, and yet they cannot speak as we do: that is, they cannot show that they are thinking what they are saying. On the other hand, men born deaf and dumb, and thus deprived of speech-organs as much as the beasts or even more so, normally invent their owns signs to make themselves understood by those who, being regularly in their company, have time to learn their language. This shows not merely that beasts have less reason than men, but that they have no reason at all […] [I]t would be incredible that a superior specimen of the monkey or parrot species should not be able to speak as well as the most stupid child – or at least as well as a child with a defective brain – if their souls were not completely different in nature to ours. (1988, 45)

For Descartes, then, the capacity for propositional language remains a product of a rational soul annexed to the body rather than any simple precipitate of the corporeal organs. It is the faculty of speech that lifts the human both beyond the animal and beyond the limits of its own flesh.

Though hardly an expert, Samuel Beckett knew more than a little about Descartes’s philosophy (see Feldman 2006, 46–57). His early poem *Whoroscope* (1930) is coddled with obscure references to Descartes, alongside the description of a “pale abusive parakeet” (Beckett 2002, 5). As is perhaps more well-known, a parrot also appears in *Molloy* (1947) that is as foul-mouthed as Jackson’s Poll:

He exclaimed from time to time, Fuck the son of a bitch, fuck the son of a bitch. He must have belonged to an American sailor, before he belonged to Lousse. Pets often change masters. He didn’t say much else. I’m wrong, he also said Putain de merde […] Lousse tried to make him say Pretty Polly! I think it was too late. He listened, his head on one side, pondered, and then said, Fuck the son of a bitch. (2009, 35–6)

It is, of course, in Beckett’s *Malone Dies* (1947–8) that a pink and grey parrot called Polly belonging to a “Jackson” appears, although this bird does not swear; instead, the “dumb companion” is taught to utter the scholastic dictum “Nihil in intellectu, etc.” (2010a, 44) – a phrase that might be translated as “nothing in the mind”. Ironically, given the fact that the subject of the paragraph is supposedly “conation” (what might be glossed as the part of the mind given over to will and volition), Malone notes that the animal is unable to get as far as “the celebrated restriction” (44) that usually accompanies the phrase: “*quod non prius fuerat in sensu*”. The joke is that the parrot is only able to utter that there is nothing in the mind at all without the usual qualifier, “that has not first been in the senses”, and also without Leibniz’s suggestion that the mind itself should be the exception to this rule.^1^ If it is true that there is nothing in the mind that has not first been in the senses apart from the mind’s own singularity, this parrot cannot articulate it; instead, it remains nailed to reproductions that, in the end, have never securely been in the mind, or not if Descartes is to be believed.

Daniel Albright has suggested that Jackson’s parrot is “a kind of ideal recording device” (2003, 91), and Beckett’s obsession both with repetitions and forms of mechanical reproduction, with language loosened from the tightened intentionality of a rational mind and a self-identical speaking subject, echoes across the *oeuvre*. Much of Beckett’s work indeed seems precisely to be exploring the possibility of understanding language beyond the Cartesian philosophical frame – beyond its underpinning of the human as the animal that talks (*anthropos zoon logon echon*) and therefore the rational animal, as Aristotle has it (1984, 1253). But if this is so, there might be another Jackson who could be brought into illuminating contact with Beckett and both his avian and rather more human parrots. For it was the neurologist John Hughlings Jackson who, in 1874, first explored in both depth and detail a mode of involuntary parroting in human speech: the phenomenon of “aphasic speech automatisms” or unwilled recurrent or recurring utterances in people who had undergone brain damage, most usually to the anterior left hemisphere. Though sufficiently influential to become known as “the father of English neurology” (Critchley 1998), there is no evidence that Beckett read Hughlings Jackson’s work, despite probably encountering the name and his theories of epilepsy when reading William Osler’s *Principles and Practices of Modern Medicine* (1895) in the early 1930s. ^2^ Beckett did, however, have both artistic and concrete encounters with aphasia (language processing impairments) and speech automatisms. In 1937, he began writing a dramatic fragment about Samuel Johnson called *Human Wishes*, and the notes Beckett took from Boswell on Johnson’s life reveal an insistent interest in the author’s illnesses and his disordered speech,^3^ alongside the temporary aphasia he experienced following a stroke in 1783 (Löwe 1999, 197). It was later, during the War, however, that Beckett was to see for himself the devastated linguistic reality of aphasia. In 1940, he briefly met and conversed, as best he could, with the poet Valery Larbaud, whom a stroke had left with nothing but the melancholy speech automatism “Bonsoir, les choses d”ici-bas” (“Farewell material things of the earth”) (Boller 2005, 85).^4^

So it is clear that Beckett had some understanding of the qualities of aphasic speech and had perhaps even read about Hughlings Jackson’s work; but we believe that Hughlings Jackson’s post-Darwinian account of language as something that emerges from various levels of representation within the brain and from a central nervous system laid down over evolutionary time, was also part of a general revolution in the conception of language itself with which Beckett’s work finds itself in clear though complex dialogue. For Hughlings Jackson brings to scientific and cultural visibility the fact that language cannot be understood as simply the expression of the willed, most abstract, or “highest” qualities and strata of the mind; it must also be understood as formed within and produced by the more “automatic”, “primitive”, “emotional” parts of an evolutionarily developed brain. Reading Beckett’s work alongside Hughlings Jackson’s thus allows us to explore the ways in which Beckett persistently returns to a sense of language as the product of a fragile, material brain which, though partially able to subserve subjective intention and rationality, is nevertheless always in continuity with a material body and in cahoots with those automatic and the involuntary aspects of human functioning that force understandings of the self beyond the Cartesian *cogito*.^5^

## Language of the borderman

Although Beckett was clearly interested in Descartes’s work, he was never simply Cartesian in either his thinking or his writing and certainly never imagined that language could be detached from corporeality. For even during the 1930s, while Beckett was plagued by a sense that no one was interested in publishing his work and that he was not in full control of his artistic material, he was able to imagine a strikingly embodied aesthetic that might make something from this incompetence. In a letter to his friend Thomas MacGreevy in 1932, Beckett indeed speaks of wanting to produce a writing that would not be “facultatif” (Fehsenfeld and Overbeck 2009, 133), optional; instead, he imagines producing an ejaculatory form of expression that would be obligatory, reflexive even: “I’m in mourning for the integrity of a pendu’s [hanged man’s] emission of semen […] the integrity of the eyelids coming down before the brain knows of grit in the wind” (134–5).

Afflicted with boils and cysts that he imagined he worked up out of his self-involvement and mental distress, Beckett here associates all of his writing that does “not represent a necessity” (Fehsenfeld and Overbeck 2009, 133) with a style that somehow mirrors his ailments, calling it “the work of the abscess” (134). Paradoxically, however, the fact that Beckett’s abscesses wept involuntarily, refusing to sit peaceably with his cognitive intentions, also seems to figure a little hope – a precious, albeit abjected, sense of relief and release from an art that otherwise felt “all frigged up, in terram, faute d’orifice [for want of an orifice]” (Fehsenfeld and Overbeck 2009, 134).^6^ Here, one sees Beckett turning his language towards modes of reflexive embodiment that might puncture the tightened, stretched skin of an artistic intention linked with cognition and the onanistic exercising of will. In *Dream of Fair to Middling Women*, which he was writing in the same period, Beckett muses, longingly, that though “[t]he night sky was stretched like a skin”, there might be some possibility of “scal[ing] the inner wall, his head would tear a great rip in the taught sky, he would climb out above the deluge, into a quiet zone above the nightmare” (1993, 27). And seven months after the letter to MacGreevy bemoaning the “work of the abscess”, he is able now to imagine the possibilities of a cystic system and of writing as an unwilled bodily emission: “It’s an ill cyst that blows nobody any good. I find it more and more difficult to write and I think I write worse and worse in consequence. But I still have hopes of its all coming in a gush like a bloody flux” (Fehsenfeld and Overbeck 2009, 159).

It was perhaps not until the *Trilogy* and *Texts for Nothing*, written in an extraordinary, compulsive burst of creative activity between 1947–1951, that Beckett was finally to find an appropriate artistic container for this aesthetic of spontaneous emission. Here, language is imaged as vomit, shit, slobber and tears that dribble or gush from his creatures. In *Text for Nothing VIII* (1951), an aesthetic that resonates with the earlier notion of a weeping abscess indeed mingles verbal outpouring with bodily fluids, words with tears, as the discrete qualities of determinable, intention-bound meaning seep towards disorganization:

I confuse them, words and tears, my words are my tears, my eyes my mouth […] it’s forever the same murmur, flowing unbroken, like a single endless word and therefore meaningless, for it’s the end that gives meaning to words. (1999, 40)

In *The Unnamable* (1949), anal incontinence has already become an analogue for the birth of language, subject and artwork in a way that oddly and explicitly repeats those feared and longed-for emissions and explosions of the 1930s: “I’ll let down my trousers and shit stories on them, stories, photographs, records, sites, lights, gods and fellow-creatures […] Be born, dear friends, be born, enter my arse, you’ll just love my colic pains, it won’t take long, I’ve the bloody flux” (2010b, 97). While the cockatoo in *Mercier and Camier* (1946) has a verbal constipation that matches its anal blockage (1974, 27), by the time *The Trilogy* is reached, parrots can and do utter. The Unnamable is force-fed and vomits forth words of others that will never be fully at one with its intentional capacities, stating:

It is they who dictate this torrent of balls, they who stuffed me full of these groans that choke me. And out it all pours unchanged, I have only to belch to be sure of hearing them, the same old sour teachings I can’t change a tittle of. A parrot, that’s what they’re up against, a parrot. (2010b, 49)

For better and for worse, writing and language pour out in both terror and relief as shit, vomit, slobber, pus and tears in a relentless logorrhea. *Nihil in intellectu* indeed.

It has often been noted that in the early 1930s Beckett was keen to hoard words, ideas and symptoms of physical and psychological pathology. The reasons why Beckett seemingly felt compelled to do this come into focus, however, when a link is made between his gathering up of signs and symbols of states of mind and corporeality that have the frisson of compact with the compulsive, with so-called mindlessness, and his search for an aesthetic practice that could write itself beyond the frame of an artistic will “frigged up” with cognitive intentions. As Ulrika Maude has suggested, some of the earliest appearances of neurological and psychological pathologies in Beckett’s work are to be found in the *Dream Notebook* of the very early 1930s (2008, 158, 160). There, Beckett transcribed from Max Nordau’s 1892 treatise on *Degeneration* terms such as “Zwangs-Vorstellung (coercive idea, obsession)”, “aboulia (absence of will)”, “echolalia (word & sound repetition)”, and “logorrhea” (Beckett 1999, 89, 89, 91, 92). But the link with aesthetic practice could never have been far from Beckett’s mind. Nordau had studied under the famous neurologist Jean-Martin Charcot and dedicated *Degeneration* to Cesare Lombroso (the psychiatrist who infamously sought to find the stigmata of mental and nervous degeneracy in the physiognomy of criminals); he was thus, at one level, profoundly non-dualist. And yet, the terms of value with which Cartesian thought is freighted are smuggled back into Nordau's work as he praises manifestations of human culture that represent control of the “higher” faculties over the “lower”. Indeed, he explicitly condemns as symptoms of pathology the modern art of the 1890s and its eschewal of the rationality associated with realism and modes of formal, figurative ordering. Disturbed by “lower” faculties that will not remain in their rightful place, modern artists are, by Nordau’s account, afflicted by a moral and nervous lassitude that it takes a physician’s eye to diagnose. He opines:

…the physician, especially if he have devoted himself to the special study of mental and nervous maladies, recognises at a glance, in the *fin-de-siècle* disposition, in the tendencies of contemporary art and poetry, in the life and conduct of men who write mystic, symbolic and “decadent” works, and in the attitude taken by their admirers […] degeneration (degeneracy) and hysteria, of which the minor stages are designated as neurasthenia. (Nordau 1895, 15)

For Nordau, then, these artists, their work and their audiences are contaminated by expressions of mind that are degenerate because defective in cognition and conation – they are compulsive, willless. As Nordau puts it, and as Beckett transcribes, “Genius”, at least of this sort, “is a disease of the nerves” (Beckett 1999, 90).

Beckett surely hoped that his own unruly bodily systems might allow him to be counted among those whom Nordau called the “high degenerates, bordermen, mattoids, and graphomaniacs” (1999, 89) of modern art. He certainly suggested to MacGreevy, in what seems like both pride and despair, that he remained compelled to reject what was high-minded in his poetry to pursue the back passages of art: the “”Give us a wipe” class of guttersnippet continues to please me”, he groans. For, as he goes on to note, “[o]ne has to buckle the wheel of one’s poem somehow […] or run the risk of Nordau’s tolerance” (Fehsenfeld and Overbeck 2009, 87) – a tolerance seemingly much worse than being classed a dirty “degenerate”. Although it is clear that there is something disgusted in this self-recognition, the “guttersnippet” becomes a kind of contamination that also offers up hope of a cure. As thought, language and will are forced into an alarmingly slippery continuity with the somatic, there is the sense of liberation in finding a language that might be detached from what is paralysingly self-conscious. Still, for an unsuccessful artist who continually felt “shatupon” by at best indifferent and at worst dismissive publishers and public,^7^ holing up with the “bordermen” is an act fraught with ambivalence. His oddly compulsive method of parroting material from Nordau allows him to be positively identified with those modern artists compelled by obsessions, pathologies, bodily drives and “coproplalia (mucktalk)” (Beckett 1999, 97), but the abjection of the method insists. For Beckett was to complain of feeling ‘soiled […] with the old demon notesnatching” (qtd. in Pilling 1999, xiii),^8^ and later, when in Germany in 1937 and filling his diary with lists of dates and prosaic details of painters and paintings, the “mindlessness” of notation becomes even more explicitly ambivalent. He affirms, at one moment, that “[w]hat I want is the straws, flotsam, etc., names, dates, births and deaths, because that is all I can know” (qtd. in Knowlson 1996, 244), and yet, only a short while later, he pitifully condemns himself as:

…pathologically limp and opinionless and consternated. The little trouble I give myself, this absurd diary with its lists of pictures, serves no purpose, is only the act of an obsessional neurotic. Counting pennies would do as well. An “open-mindedness” that is mindlessness, the sphincter of the mind limply forever open, the mind past the power of closing itself *to everything but its own content*.

*I have never thought for myself*. (qtd. in Knowlson 1996, 252)

Caught between the release and the abjection of mindlessness, Beckett indeed seems compelled to experience both the pleasures and pains of the “borderman”.

Nordau spoke, in fact, of what “Maudsley and Ball call […] ‘Borderland dwellers’ – dwellers on the borderland between reason and pronounced madness” (1895, 18); but perhaps it is more suggestive to think of the border territory as that marked out between what is willed by a fully intending mind and what is simply automatic. For despite his clear interest in involuntary emissions, Beckett was never to be engaged by the fluid transcriptions of unconscious states produced in so-called “automatic writing”. In 1930, while lecturing at Trinity College Dublin, he affirmed that pure unconscious states should not be used in literature as they “destroy the integrity of the real” (qtd. in Le Juez 2008, 53). Rachel Burrows’s lecture notes record instead Beckett’s approving remarks on the forms of half-consciousness the author of *Degeneration* found so troubling: “Gide interested in liminal consciousness (sneered at by Nordau)” (qtd. in Pilling 1999, 91), Beckett told his students. These assertions are revealing because in his own aesthetic work that followed, Beckett was insistently to imagine language in terms of bodily functions that, though hardly expressions of an intending mind, are rarely fully automatic. The body’s oozings and excreta are, instead, more accurately understood to be highly susceptible to the formation of habits that can subserve a person’s purposes, even as they always bear the threat of an uncontrolled emission. Of course, the fact that these bodily functions are plastic, malleable in relation to the mind, produces problems for a *cogito*. The Beckett who read psychoanalytic work in detail, fearing he was an “obsessional neurotic” (Fehsenfeld and Overbeck 2009, 273), certainly lived out the terrible failure of Cartesian dualism in the irruptions of the psychosomatic: he experienced the appalling tendency for seemingly mental states to convert themselves into physical symptoms and for physical problems to produce mental distress. And yet, he came to produce an aesthetic somehow aligned with his powerfully disrupted peristalsis and with what a doctor once described as his “deep-seated septic cystic system” (Fehsenfeld and Overbeck 2009, 144). Although it is clear that he hopes that various forms of somatic “incontinence”, transformed into language, might cure his writing of its blockages, from its constipating intentions, what Beckett seems truly compelled by is not a language that is completely mindless but one that bears witness to the compact between mind and body, intellection and emotion, between the intention and the automaticity both in and of words. It is precisely in this “borderland”, we maintain, that Beckett’s psychosomatic language stakes its aesthetic claim.

## Function running away with organ

If one were searching for representations of the automatic in Beckett’s work, one might well alight on *Not I* (1972), where the speaking subject is reduced to a mouth isolated in the blank blackness of the stage space, spitting and leaking language. Mouth experiences herself as subjected to the jagging, gagging and glitched fastforwards and rewinds of mechanisation, and although her words emerge from the matter inside, from a “dull roar in the skull”, their source is also somehow beyond her:

…all dead still but for the buzzing … when suddenly she realized … words were … […] words were coming … a voice she did not recognize … at first … so long since it had sounded … then she finally had to admit … could be none other … than her own … certain vowel sounds … she had never heard. (Beckett 1990, 379)

Despite the fact that her language emerges as a bodily emission – “nearest lavatory … start pouring it out … steady stream” (Beckett 1990, 382) – her sense of her own experience affirms that language does not emerge with absolutely unfettered unconsciousness, just as the drafts of the manuscript prove decisively that the piece was not written automatically. Mouth’s language is hardly synonymous with intention and volition, nor is it the precipitate of conation; nevertheless, the compulsive push and pull that produces words spilling over with subjective affect demonstrates that mind is far from absent from the scene.

In 1972, Beckett does seem to affirm a certain automatism within *Not I* by writing to Alan Schneider that there is a distinction to be made

…between mind & voice […] Her speech a purely buccal phenomenon without mental control or understanding, only half heard. Function running away with organ […] I hear it breathless, urgent, feverish, panting along, without undue concern with intelligibility. Addressed less to the understanding than the nerves of the audience which should in a sense *share her bewilderment*. (Harmon 1998, 283)

But the nuances of this position come more clearly into focus if one realises that, consciously or not, Beckett is parroting material here that he read nearly forty years earlier. In 1934–5, distressed to the point of incapacity by his psychosomatic symptoms, Beckett sought psychotherapy from Wilfred R. Bion and supplemented his treatment with readings on psychology and psychoanalysis. These further “notesnatchings” speak to his growing interest in the automatic and the seemingly mechanical aspects of psychology, in “behaviorism”, for example; but Beckett also transcribes from Karin Stephen’s *Psychoanalysis and Medicine: A Study of the Wish to Fall Ill*, not just “bewilderment”, but the ‘s*heer terror* of being run away with by a bodily function” (emphasis added; qtd. in Feldman 2004, 310) – the dread that can accompany what emerges without volition (Beckett 1934-5a, 2). Nearly forty years after first reading it, and perhaps surprisingly, Beckett finds himself echoing a psychoanalytic mode in his efforts to evoke a language that would bear witness to the slippery continuum between idea and matter, mind and body. This language is addressed to and perhaps more of “the nerves” than “mental control or understanding”; nevertheless, in its evocation of a form of nervous bewilderment, both character and audience find themselves pushed and pulled by affective states that refuse the relief and reflexive simplicity of any pure mindlessness.

Now, Stephen’s book is distinctive in terms of psychoanalytic texts in its emphasis on specifically embodied symptoms that are nevertheless understood to be psychogenic in origin. By the 1930s, psychoanalysis as a discipline had moved away from its neurological beginnings, from Freud’s early study of aphasia and his somewhat later sense of the material action of the nerves in the production of neurosis. Stephen’s work, however, decisively takes psychoanalysis back to the body and to the mind’s continuum with it – to the early Freud’s emphasis on the conversion of mental states into embodied symptoms according to a mysterious, almost magical sense of the body’s neurology functioning through various modes of representation and symbol formation.^9^ While psychoanalysis’s insistence upon physical symptoms as expressions of repressed ideas and stuttering, unexpressed affect worked to liberate patients from the stigma of biological “degeneracy” (Wilson 2004, 4), something perhaps also got lost in the more or less explicit disavowal of the neurological mechanisms of conversion of *psyche* into *soma*, *soma* into *psyche* (see Wilson 2004, 1–14).

Karin Stephen is similarly silent about the neurology of conversion, but she nevertheless insists on its effects. She affirms that psychogenic symptoms are neurotic defences that vent pent up nervous energy and prevent anxiety from developing when earlier acts of repression begin to falter. By her account, many such symptoms appear in relation to the repression that can attend the infant’s first experiences of ingestion and excretion and the inevitable disappointment of its natural hopes for fulfilled desire and omnipotence. As Beckett transcribed: “All psychogenic illness proceeds form [sic] deadlock between infantile sexuality & fear, aggression & rage” (qtd. in Feldman 2004, 309) (Beckett 1934-5a, 1); for if the desire and aggression that meet disappointment is not tolerated and contained by benign parental attention, the ensuing repression can produce an unconscious, seemingly involuntary, but hardly mindless hoarding and then expulsion of bodily fluids. Beckett notes down the “[o]verwhelming quality of infantile excretory processes”, which means that “they are both dreaded & desired by the subject”. These processes

…constitute a diffuse form of orgasm, taking control of the organism & carrying themselves through to a crisis independently of volition. This typical of sensation of excretion before sphincter control has been established. The child punished for lack of control may grow up dreading loss of control on various planes, excretory, genital, etc., resulting in constipation, frigidity, etc. The sheer terror of being run away with by a bodily function. (qtd. in Feldman 2004, 310) (Beckett 1934-5a, 2).

Stephen is quite clear that words frequently come to function as the instruments of this arm-twisting “deadlock”: “[p]atients often equate a flow of words or a flood of tears with excretory gifts” (1933, 114), while the “almost reflex inhibition” that results from this ‘sheer terror of being run away with by bodily function” (149), “may produce disturbances of a variety of bodily functions: speech may be checked, producing a stammer; the free evacuation of the bowels or bladder may be interfered with” (150).

The psychosomatic illnesses that Stephen describes that are driven by neuroses – by nervous matter working in concert with the mind to produce mental and somatic experience – remain under the sway of what Beckett transcribed from Ernest Jones’s *Treatment of the Neuroses* during the same period as “*Zwang*”. Laplanche and Pontalis gloss *Zwang* as denoting, in Freudian vocabulary, “a constraining internal force. It is most frequently employed in the context of obsessional neurosis, where is implies that the subject feels obliged by this force to act or think in a particular way and that he struggles against it” (Laplanche and Pontalis 1988, 77). Jones describes, and Beckett transcribes, such obsessional, compulsive symptoms in these terms:

Obsessional neurosis (Zwangsneurose): feeling of mustness. Symptoms: (1) Motor: Zwangshandlungen (avoiding cracks in pavement, etc.) (2) Sensory: [sic] (3) Ideational: Zwangsvorstellungen. (4) Affective (obsessive emotions). Also tics (habit spasms). The Zwang may appear as paralysis of the will, e.g. paralysis at the most trifling dilemma. (qtd. in Feldman 2004, 342) (Beckett 1934-5b, 23)

As we have seen, “Zwangs-Vorstellung (coercive idea, obsession)” held alongside “aboulia (absence of will)” both appear in the *Dream* notebook; but Beckett explicitly learns from Jones that this “feeling of “mustness”” strips back the subject’s capacity for intentional thought, as the “patient oscillates between the two conditions of not being able to act or think (when he wants to) and being obliged to act and think (when he doesn’t want to)” (Jones 1920, 195). Seemingly recognizing himself as compelled by *Zwang*, Beckett writes to MacGreevy after his psychotherapy had come to perhaps a precipitate end: “As I write, think, move, speak, praise & blame, I see myself living up to the specimen that these 2 years have taught me I am. The word is not out before I am blushing for my automatism” (Fehsenfeld and Overbeck 2009, 300). The automatic may offer some relief from the thinking subject but, for Beckett, psychoanalysis has seemingly taught him that what erupts in this manner has little of the relief of the knee jerk. The compulsive is not an escape from the mind; rather, it remains contaminated with and indeed driven by feeling. What emerges compulsively constellates within an affective, affected subject that remains sufficiently conscious to act as its own sometimes amused, sometimes melancholy witness.

Although to contemporary eyes the discourses of psychoanalysis and neurology may seem incommensurable, their history tells a different story. Beckett certainly read across them both in the 1930s, persistently alighting on the places where the mind’s material functioning and the body’s capacity both to receive the impression and dramatize the expression of psychological states, are brought to consciousness. In what follows, we suggest that Beckett’s determination that his art should use the emotional and the somatic to write language beyond any simple expression of intention and rationality can be historically contextualized and its nuances illuminated by placing it alongside research on aphasia. Aphasiology describes how much of our speech activity is not under ongoing, moment-to-moment control. We suggest that by exploring this meeting within speech and language of the voluntary and the involuntary, the rational and the emotional, the conscious and the unconscious, the representational and the modal, we can perhaps better understand Beckett’s vital attempt to write the linguistic self as an insistently psychosomatic entity.

## Old style

In terms of the scientific understanding of language, 1861 marks a moment when the concept of language that subtended Cartesian dualist accounts of the relationship between mind and body underwent an authentic paradigm shift. 1861 is the year that Paul Broca delivered a paper in Paris detailing the case of a patient called Leborgne who had become known simply as “Tan”. “Tan” had earned his name because, apart from a few oaths and swear words, the reiterated nonsense syllables of “*tan tan*” were the only ones he could utter, despite seemingly unimpaired intelligence and speech organs unaffected by paralysis (see Tesak and Code 2008, 46–54). Although his disability had been somewhat mysterious to his doctors, when his brain was examined by Broca at autopsy a determining cause for Leborgne’s aphasia was found. His brain was revealed to have a lesion in the third frontal convolution of the left hemisphere caused by a cyst, and this area was consequently inferred by Broca to be the seat of “the faculty of articulated language”.^10^ The third frontal convolution is now known as Broca’s area, with the broad severe form of aphasia that can include speech automatisms, nonfluency and syntactic impairments with relatively intact comprehension, given the name Broca’s aphasia.^11^ But what Broca’s work on aphasia seemed decisively to demonstrate was that, *contra* Descartes, those seemingly immaterial faculties of the human had a material seat that was linked to their causation rather than simply marking their location. Becoming the first fully materialist model of language production to be accepted as scientific orthodoxy, Broca’s contributions to aphasiology might thus be understood as inaugurating a crucial moment in the history of subjectivity. They mark the moment when key aspects of the psyche – its ability to speak, think, and form rational, abstract intentions – are shown scientifically to be dependent on an embodied, material organization (see Jacyna 2000, 3). This is also the moment that language comes to be understood as a properly psychosomatic entity.

A little later in the nineteenth century, the neurologist John Hughlings Jackson developed an idea that was to have a significant impact on aphasiology and subsequent developments in neurolingusitics. He determined that all language was more or less “propositional” in nature – dependent on context. From his observation and treatment of aphasic people, however, Hughlings Jackson also proposed the significant insight that a great deal of our spoken language is in fact produced automatically, and that this nonpropositional language arises from more primitive and ancient areas of the brain. Hughlings Jackson had turned his attention specifically to the striking phenomenon of ‘speech automatisms”, or recurring utterances, articulated mainly by people with Broca’s aphasia either frequently or invariably when they try to speak. Although these automatisms sometimes consist of nonsense syllables (*nonlexical* speech automatisms such as “*tan tan*”), one finds very commonly *lexical* speech automatisms that are made up of recognizable words and syntactically correct structures (for example, Leborgne’s other automatism, the oath “sacré nom de Dieu”). What Hughlings Jackson proposed, following the work of the evolutionary psychologist Herbert Spencer, was that language had evolved inseparably with the central nervous system. He suggested, in terms that can still be found in contemporary neurology, that language is represented at different anatomico-structural levels in the brain with expression by older levels lower down the hierarchy inhibited by younger controlling mechanisms higher up the nervous system – the highest level being the neocortex (see Franz and Gillett 2011). It was on the basis of this evolutionarily subtended model that Hughlings Jackson inferred that the kinds of phrases such as oaths and swear words that are preserved as speech automatisms after brain damage suggest “a loss of intellectual (the more voluntary) language, with persistence of emotional (the more automatic) language” (Hughlings Jackson 1884, 1).

Hughlings Jackson concluded that the nonpropositional speech he observed in aphasic speech automatisms is preserved because it is produced at lower levels in the brain and somewhat automatically. Nonpropositional speech includes cursing, swearing, rote learnt activities such as automatic counting, nursery rhymes and prayers, clichés and idioms such as “now and then” or “by the way”. The linguistic elements within such speech are not newly or individually generated in each utterance, unlike in propositional speech where original ideas are encoded into newly constructed and novel utterances. Using terms and models that are still to be found in the contemporary neurological model, Hughlings Jackson suggested that if propositional, or referential speech is under conscious control and non-propositional speech is the product of phylogenically earlier, less evolutionarily developed processes, when higher areas of brain function (such as Broca’s area) are damaged to the degree that they fail to inhibit the behaviour of the lower levels, the result can be the automatic production of emotionally charged utterances. He describes the nonpropositional qualities of oaths in these terms:

Although oaths differ from mere alterations of tone, in that they consist of *articulate words*, they are generally used in talking, not to express ideas, but to make up by vigour in delivery what is wanting in precision of expression. They may, indeed, be considered as phrases that emotion has filched from the intellect, to express itself in more definite terms than it could do by mere violence of tone or manner. (1864, 40)

For Hughlings Jackson, speech automatisms, like the oaths they retain, “in spite of their propositional structure, [have] no propositional function. No man intends to say, nor is believed by others to say, anything when he swears to his own eyes” (ie, “Cor Blimey” from “God blind me”) (1932, 135). Consequently, for Hughlings Jackson such ejaculations “take low rank in language, little above that of other bodily starts” (1880, 139). And yet, though such ejaculations are in some senses reflexive, the emotional qualities Hughlings Jackson emphasizes demonstrate clearly that such seemingly automatic language is hardly mechanical, for it is frequently and vitally concerned with the social and emotional aspects of communication. Even so, for Hughlings Jackson, and in terms that Nordau echoes, brain damage represents a kind of evolution in reverse: a “reduction to a more automatic condition: in each [case of aphasia] there is Dissolution, using this term as Spencer does, as the opposite of Evolution” (1879, 111).

Placed alongside the historical and disciplinary context of this embodied neurological understanding of language, we can perhaps understand a little more of the force of Beckett’s irony in the Unnamable’s demands for reflexive, rote learning from Mahood, just before evolution in reverse takes him back to Worm and language as slobber – reflex-driven matter:

Pupil Mahood, repeat after me, Man is a higher mammal. I couldn’t. Always talking about mammals, in this menagerie. Frankly, between ourselves, what the hell could it matter to pupil Mahood, that man was this rather than that? Presumably nothing has been lost in any case, since here it all comes slobbering out again. (2010b, 50)

In a similar vein, Ulrika Maude has demonstrated Beckett’s characters’ propensity for ticcing obscenities, for the kind of coprolalic “mucktalk” found in Tourette’s syndrome (2008, 160) that is produced, in neurological terms, by the disinhibition of language linked to urges that are most likely associated with the phylogenically earlier basal ganglia of the brain (Nespoulous et al. 1998, 329). But Beckett’s insistent, albeit untheorised concern with precisely the forms of language so oddly preserved in speech automatisms is perhaps even more clearly to be found in his stylistic obsession with the manipulation of idiom and cliché. As Elizabeth Barry has meticulously shown, in Beckett’s texts smithereens of readymade language work as expressions of a kind of “collective memory” (2006, 65) that precisely interfere with an idea of art as the willed recollection and then expression of individual subjective experience. As she puts it, Beckett’s persistent stylistic return to cliché, aligned with the *Trilogy’s* thematic account of language as the eruption of improperly digested sustenance – vomit and the “bloody flux” – shows how language has the capacity to be “regurgitated automatically without being assimilated to the self” (89). Similarly, Winnie in *Happy Days* (1961), though certainly capable of uttering more than “automatisms”, nevertheless remains strikingly nailed to the same nonpropositional language that finds itself preserved in aphasic speech automatisms – rhymes, prayers, clichés and idioms. The very first lines of the play set this language in operation, as Winnie, in evening dress though buried in a mound up to her waist, intones: “Another heavenly day […] *Lips move in inaudible prayer* […] For Jesus Christ sake Amen […] World without end Amen” (Becket 1990, 138). Surrounded by “old things”, by the detritus of a life she can no longer inhabit, she remains incapable of finding a new style of speaking that might suit her situation. Instead, sensing the merest flicker of comfort within it, she rests on the language of half-remembered clichés (“what are those wonderful lines” (Beckett 1990, 140)) and the “old style” (143) to which she persistently returns and upon which, through which, she lies. Although Winnie is presumably speaking historically when she refers to the “old style”, it is highly suggestive that Billie Whitelaw, when attempting to find a voice in which the later play *Not I* could be uttered, spoke of an intuitive need to return to the regional accent of her childhood – one might say an ontogenic “old style” – rather than the received pronunciation she acquired later at drama school.^12^ Aphasiologists have suggested that the remarkably restricted semantic range of lexical speech automatisms may indicate a common limbic basal neurogenic location for this nonpropositional language (see Code 1982, 149); and what aphasiology thus allows us to map in Beckett’s work is the extraordinarily persistent desire to invoke forms of language that seem both phylogenically and ontogenically to precede the propositional language we associate with a the “higher” functioning of an intentional consciousness.

The final link we wish to draw between aphasic speech automatisms and Beckett’s mature style is the propensity for using the most commonly observed aphasic lexical automatism – pronoun plus modal or auxiliary verb constructions. Lexical speech automatisms of this sort rarely break syntactical rules, but they are commonly emotionally toned and incomplete or interrupted before any main verb can be evoked (I can’t … I want to) (see Code 1982; Code et al. 2009, 446). Now, the evacuation of main verbs, alongside the suggestive figuration of what is left as bodily discharge, oddly insists in Beckett’s texts, functioning as a stigmata of linguistic incompetence. In *Rough for Theatre II*, the bureaucrat reading the report of a potential suicide’s life shouts:

B: Shit! Where’s the verb?

A: What verb?

B: The main!

A: I give up.

B: Hold on till I find the verb and to hell with all this drivel in the middle. [*Reading*.] “… were I but … could I but …” –Jesus!– “…though it be … be it but” –Christ! –ah! I have it– “… I was unfortunately incapable…” Done it! (1990, 243)

In *Text for Nothing II*, a similar evacuation of main verbs is invoked, only here there seems to be a more or less explicit link with a sense of damaged matter in the head, the skull: “it must be in the head, slowly in the head the ragdoll rotting, perhaps we’re in a head […] ivory dungeon. The words too, slow, slow, the subject dies before it comes to the verb” (1999, 13).

The most ubiquitous feature of aphasia with which these examples seem to resonate is *anomia*: the failure to access a lexical item, but most commonly nouns or verbs (see Laine and Martin 2006). As Hughlings Jackson has shown, however, not all language is affected equally. While representational language is severely impaired in aphasia, what have been termed “modal” structures remain more easily accessible. Of course, within non-pathological speech modal verb constructions commonly mark out the territory of subjective possibility: I must, I shall, I will, I should, I would, I can, I could, I may, I might. And in a general sense the *Trilogy* finds itself definitively within the terrain of such modal verbs: probability, ability, obligation and advice, permission, habits. Towards the end of *The Unnamable*, the pronoun plus modal/auxillary verb construction indeed begins insistently to appear, though, crucially, in a negative form. As the desperate, futile attempts to articulate a voice which isn’t simply the voice of others, or otherwise give up on speaking completely, are ratcheted up, “I” (the most common word in the largest corpus of aphasic speech automatisms compiled in English (see Code 1982) begins to cascade down the page, with comma splices invoking a sense of both interruption and urgent propulsion:

I must understand, I’m doing my best, I can’t understand, I stop doing my best, I can’t do my best, I can’t go on, poor devil, neither can they, let them say what they want, give me something to do, something doable to do, poor devils, they can’t, they don’t know, they’re like me, (2010b, 103)

Propositional, referential, language seems on its way to disappearance, to be replaced with a rhythmic flow of modalizing:

…you must say words, as long as there are any, until they find me, until they say me, strange pain, strange sin, you must go on, perhaps it’s done already, perhaps they have said me already, perhaps they have carried me to the threshold of my story, before the door that opens on my story, that would surprise me, if it opens, it will be I, it will be the silence, where I am, I don’t know, I’ll never know, in the silence you don’t know, you must go on, I can’t go on, I’ll go on. (2010b, 134)

Here, though, as in the aphasic speech automatism, the modal verb appears in a form that precisely suggests incapacity and uncontrolled, compulsive necessity, rather than possibility.

One of the things an understanding of aphasia allows us to tease out in Beckett’s texts is that although the modal language that begins to dominate towards the end of *The Unnamable* may not be the language of a *cogito*, it nevertheless retains a sufficient link with consciousness to disallow any ecstatic, unfettered automaticity. For modalizing language constellates precisely around subjective attitude and social demands, and although aphasic speech automatisms may be more automatic than not, they are always formed in dialogue with capacities and articulations of consciousness that are retained. Indeed, this non-propositional language emerges because of a failure of the aphasic person’s “executive functions” (which include attention, shifting between mental sets or tasks, updating/monitoring of contents of working memory, and awareness and inhibition of prepotent responses) and disinhibition of the “lower” strata of language ensues. Consequently, not only are emotional functions preserved, they can also appear to be heightened with the loss of inhibition. Aphasia seems, in fact, to be a disorder that often leaves the capacity of the self to feel and gauge its losses within consciousness agonisingly untouched, with speech automatisms seeming to appear with greatest potency precisely when aphasic people are asked to deploy their impaired executive functions in speaking (Code et al. 2009, 454–5). The incapacity, inability and frustration produced by a sense of demand – a demand for answers or for referential and propositional speech that Beckett also persistently dramatizes – indeed seems to release this automatic, modalizing language. When left hemisphere damage removes any possibility of propositionizing, modalizing language, which appears to be produced by both right and left hemispheres, begins to utter in anxious compensation. Not only is this the only language to which most people with aphasic speech automatisms have access, it is the language that begs to be expressed as a reaction to the failure to access propositional speech (Nespoulous et al. 1998, 323–4). Clinical work with aphasic patients indeed seems to suggest that the frustrated incapacity of “I can’t” is precisely linked to the production of words under “I must” conditions – conditions that release, even propel them.

Beckett famously asserted to Lawrence Harvey the sense of compulsion that seemed to subtend his artistic production: “I write because I have to […] What do you do when “I can’t” meets “I must”?” (Harvey 1970, 249). Beckett’s texts indeed seem to dramatize a compulsive back and forth in this border territory triangulated between the unreachability of full linguistic propositionality and an absolute automaticity or indeed a silence that can never finally be achieved. The words that insist in this borderland instead refract what remains of the speaking self through both the idea and the sense of a subjectivity it can no longer inhabit, and an unfettered unconsciousness it knows it cannot attain. So in his search for words that could be held in this territory Beckett turns, albeit in an untheorised fashion, to a modalizing language imaged as a body language of slobber, pus, shit, tears and vomit. Although this is a language that necessarily sticks in the craw of the idea of speech as an unsullied expression of cognition and intention, it is a language that uses both the “understanding” and the “nerves” of a reader and audience to stage the fact that *zoon logon echon* is never fully conterminous with the rational animal.

## Between can’t and must

As he came towards the end of his life, Beckett struck up a friendship with a writer called Lawrence Shainberg who was exploring neurological dysfunction in his work. And it was in his conversation with Shainberg that Beckett himself makes a link between a writing compulsively held between “can’t and must” and neurological dysfunction. As Shainberg recounts, Beckett affirmed that

“With diminished concentration, loss of memory, obscured intelligence — what you, for example, might call ‘brain damage’ — the more chance there is for saying something closest to what one really is. Even though everything seems inexpressible, there remains the need to express […]”. Of course, he knew that this was not a new project for him, only a more extreme version of the one he’d always set himself […] It was always here, in “the clash,” as he put it to me once, “between can’t and must” that he took his stand. (1987, 103)

Although these conversations took place towards the end of Beckett’s life, they seem to gesture backwards. For one can hear in them what Beckett described to Georges Duthuit, during the time he was writing *The Unnamable,* as the need to find a “non-relational”, a disconnected art. He states that the artist should be “at ease enough with the great tornadoes of intuition, to grasp that the break with the outside world entails a break with the inside world, that there are no replacement relations for naïve relations”. Art must indeed, in Beckett’s terms, instantiate “the impossibility of reconnecting” (Craig et al. 2011, 140), and he goes on to affirm that “to want the brain to function is the height of crassness” (149). The idea that Beckett might somehow be using a mode related to neurological dysfunction and disconnection comes even more clearly into focus in his suggestive remark to Shainberg that the writing he developed during the *Trilogy* emerged from an affectively charged encounter with Parkinson’s disease. Shainberg recounts:

…speaking of *Molloy* and the work that followed it, he told me that, returning to Dublin after the war, he’d found that his mother had contracted Parkinson’s Disease. “Her face was a mask, completely unrecognizable. Looking at her, I had a sudden realization that all the work I’d done before was on the wrong track. I guess you’d have to call it a revelation. Strong word, I know, but so it was. I simply understood that there was no sense adding to the store of information, gathering knowledge. The whole attempt at knowledge, it seemed to me, had come to nothing. It was all haywire. What I had to do was investigate not-knowing, not-perceiving, the whole world of incompleteness”. (1987, 105)

In the face of neurological damage, Beckett does not turn away from language; rather, he turns towards a new kind of work that disarticulates language’s seemingly immutable connection with knowledge, intention, propositionality and the rational self-expression of a *cogito*. In another interview, Beckett affirmed again his orientation towards “the whole world of incompleteness”, stating: “I’m no intellectual. All I am is feeling. ‘Molloy’ and the others came to me the day I became aware of my own folly. Only then did I begin to write the things I feel” (D’Aubarède 1979, 217). And perhaps by noting the suggestive symmetry between the language preserved in the aphasic speech automatism and some of the central features of Beckett’s postwar work, we can gain some understanding of how and where, in neurological terms, Beckett was to find his compulsive, decidedly un-Romantic language of feeling, and how and where it continues to rattle and resonate within readers and audiences.

The aphasiologist Théophile Alajouanine, when writing in the 1940s of the aphasic poet Valery Larbaud whom Beckett met during his illness, is clear that Larbaud’s inability to speak and write propositional language fundamentally disarticulated the possibility of the author’s literary production. For Alajouanine, aphasia “abolished the possibility of literary artistic realization” for Larbaud, although he goes on to add this qualifier, “at least the one he was used to for he did not employ that conventional agrammatism of some of the young literary schools” (1948, 231). Presumably, Alajouanine means that Larbaud never had written, so never could write, like a Joyce (whose *Ulysses* he translated), a Surrealist, or one of Nordau’s “degenerates”. As far as Alajouanine is concerned, “[t]he aphasic patient, with his involuntary use of ready-made sentences unadapted to what he wants to express, shows in some way a negative picture of literary art” (239). But Beckett, who was well-versed in modernism’s attachment both to agrammatism and to “readymades” is no longer seeking “naive relations” in his art; he does not wish for the brain and the writing hand simply to function with the “crassness” of the normative. Perhaps in his search for a non-relational art of “not-knowing”, of “incompleteness”, Beckett was to intuit, through its affect, something of the functional dissociation between propositional-referential and modalizing language that neurolinguistic understandings of language have revealed, invoking such disturbance as a method that might precisely enable his aesthetic bound to buckling the wheel of the word. Staked to and compelled by the affective space between “I can’t “and “I must” of modalizing utterances spewed, spat, dribbled and shat out, Beckett seems to find a language that beggars the putative power of knowledge and intentional capacity, while retaining a sense of an affective life that hopes for expression and to speak for itself even amidst its decomposition. The Samuel Beckett who returned obsessively to the glitches and discontinuities in and of translation, to the impossibility of smooth conversions between media, the Beckett who compulsively refused the naive relations between languages, genres and, perhaps most fundamentally, between mind and body – breaking up and breaking down the imagined simplicity of the continuum between them – seems indeed to offer us the beginnings of a new mode of reading and experiencing. He offers us the opportunity both to comprehend and to feel how language drives the torsions and vicissitudes of our psychosomatic lives.

## Endnotes

^1^ Beckett noted down the dictum from Wilhelm Windelband’s *A History of Philosophy* (1931) in his “Whoroscope Notebook” (1932–7), but included Leibniz’s response to Locke’s empiricism: “*nisi ipse intellectus*” [except the mind itself].

^2^ A number of entries from Osler’s work are transcribed into Beckett’s *Dream Notebook*. Adam Winstanley (2013) has suggested a link between Osler’s accounts of Hughlings Jackson’s work on epilepsy and the convulsive narration and verbal outbursts in *Molloy*.

^3^ Laura Davies suggests that many doctors now believe that Tourette’s syndrome is the most likely explanation for Johnson’s symptoms (2014, 47).

^4^ Knowlson (1996, 298–9) offers an account of the meeting between Beckett and Larbaud, while Francois Boller (2005, 85) gives details of Larbaud’s aphasia. The translation of Larbaud’s automatism is Boller’s, although he concedes that it “has a literary flavour, which is difficult to translate”. The phrase is idiomatic, in a rather archaic and figurative way.

^5^ A number of critics have explored the link between Beckett’s work and neurological understandings of language subtended by studies of aphasia. See Beausang, Salisbury, Hubard, Laranjinha.

^6^ LS believes that the editors of the published version of this letter have mistranscribed “frigged” as “trigged”, and “in terram” as “in terrain”.

^7^ In a letter of 1934, Beckett called Chatto and Windus, who were rather desultorily publishing *More Pricks than Kicks*, ‘shatupon & Windup” (Fehsenfeld and Overbeck 2009, 212).

^8^ Undated letter to Thomas MacGreevy probably written in early August 1931.

^9^ For an account of the relationship between the aphasiological, neurological and psychoanalytic Freud and the importance of language in his thought see Forrester (1980).

^10^ The qualification “articulated language” was important, as damage to other areas of the brain were identified later in the century as apparently responsible for other forms of aphasia, including significant language comprehension impairments – *sensory* aphasia.

^11^ Broca himself preferred the term *aphemia* as describing better the significant problems with the organization and production of articulated speech, despite a lack of paralysis affecting the speech organs, he identified.

^12^ We are grateful to Mary Bryden for sharing this illuminating detail, which she received in conversation with Billie Whitelaw.
